# Beneficial Effects of Oral Nutrition Supplements on the Nutritional Status and Physical Performance of Older Nursing Home Residents at Risk of Malnutrition

**DOI:** 10.3390/nu15194291

**Published:** 2023-10-08

**Authors:** Yi-Hsiu Chen, Che-Yu Lee, Jiun-Rong Chen, Min-Yu Ding, Feng-Qi Liang, Suh-Ching Yang

**Affiliations:** 1 School of Nutrition and Health Sciences, Taipei Medical University, Taipei 11031, Taiwan; ma07108007@tmu.edu.tw (Y.-H.C.); ga56107001@tmu.edu.tw (C.-Y.L.); syunei@tmu.edu.tw (J.-R.C.); 2 Chia-Ying Nutrition Consultation Center, Taipei 10068, Taiwan; dmy1223@gmail.com (M.-Y.D.); vicky28mm@gmail.com (F.-Q.L.); 3 Research Center of Geriatric Nutrition, College of Nutrition, Taipei Medical University, Taipei 11031, Taiwan; 4 Nutrition Research Center, Taipei Medical University Hospital, Taipei 11031, Taiwan; 5 School of Gerontology and Long-Term Care, College of Nursing, Taipei Medical University, Taipei 11031, Taiwan

**Keywords:** malnutrition, older nursing home residents, physical performance, oral nutritional supplement, nutritional education

## Abstract

The purpose of this study was to compare the effects of nutritional supplement drinks (NSDs) and nutritional education (NE) on the nutritional status and physical performance of older nursing home residents who were at risk of malnutrition. This study was a clustered, randomized, parallel, multi-center clinical trial, with 107 participants more than 65 years old and at risk of malnutrition recruited from several nursing homes in this study. Participants were divided into two groups: an NE group (*n* = 50) and an NSD group (*n* = 57). The NE group was given NE by a dietitian, while the NSD group was provided with two packs of NSD except receiving NE (Mei Balance, Meiji Holdings, Tokyo, Japan) per day as a snack between meals and before bed. Anthropometric data, blood pressure, nutritional status, blood biochemical biomarkers, and physical performance were measured before and after 12-week interventions. After 12 weeks of NE combined with NSD intervention, body weight, body-mass index, the mini nutritional assessment-short form (MNA-SF) score, walking speed, and SF-36 questionnaire score were improved in older nursing home residents at risk of malnutrition.

## 1. Introduction

The World Health Organization (WHO) estimates the total number of older or older people (≥60 years) worldwide will surpass 1.2 billion by 2025 [1]. Malnutrition is a common health problem in older individuals, which is related to decreased protein and energy intake and is attributed to sarcopenia, frailty, chronic diseases, a decreased ability to chew and swallow, etc. Moreover, protein-energy malnutrition can affect physical performance and muscle loss, increase incidences of falling and hospitalization, and the mortality rate in the older population [2,3].

The WHO reported that the prevalence of malnutrition in older people ranges from 1.3% to 47.8% [4]. A systematic review also showed that the prevalence of malnutrition and at risk of malnutrition were 11.7–60% and 21–60% in older people, respectively [5]. According to research in the US, the prevalence of malnutrition was 1~15% in older persons who lived in the community, but that increased to 25~85% among those in nursing homes and hospitals [6]. Those studies showed that residents in nursing homes had a higher rate of malnutrition than community-dwelling older adults. A study in Taiwan showed that around 3.6% of community-dwelling older adults were in a state of malnutrition [7]. Nevertheless, the Nutrition and Health Survey in Taiwan in 2013–2016 reported that approximately 40% of older adults had insufficient intake of energy, while 18.2% of older adults did not reach 75% of the recommended protein intake [8]. The reports indicated that the prevalence of malnutrition in older adults was rising. Taiwan became an aging society in 2018, which meant that 14% of Taiwanese were older than 65 years. It has become increasingly important to improve the health of older adults in a simple and easy way because of the growing senior population, the elongation of life expectancy, and the lack of caregivers [9].

Oral nutritional supplements (ONSs) can provide high-quality nutrients and are easy to prepare, and they are an ideal choice to provide protein and energy in patients and older individuals [10]. Recent studies reported that ONSs can be a strategy for nutritional interventions for inhibiting body weight (BW) loss and accelerating wound healing because of elevated total nutrient intake [11,12]. Moreover, ONSs had positive effects on mild and moderate sarcopenia in older persons, including increasing gait speed and muscle strength [10,12]. On the other hand, nutritional education (NE) can also increase energy intake through changing a subject’s eating behaviors [13]. NE is a time-saving and cost-effective way to improve nutrition knowledge and behaviors among older diabetics and those with other nutrition-related conditions [14]. Studies reported that older people decrease their dietary intake due to influences of social and physiological changes, including changes in taste and chewing and swallowing abilities, and these can reduce the overall nutritional status [14,15]. Although dietary educational interventions can improve people’s dietary variety, nutritional status, and health-related eating behaviors, their efficacy is limited in target populations of older adults [16].

Several studies demonstrated the unique effects of ONSs in improving the nutritional status of older adults, but most studies focused on the late stage of malnutrition [17,18]. Moreover, there is limited research on ONSs as an intervention for older adults at risk of malnutrition, especially older nursing home residents [6,19]. Therefore, the purpose of this study was to investigate the effects of nutritional supplement drinks (NSD) on the nutritional status and physical performance of older nursing home residents who were at risk of malnutrition.

## 2. Materials and Methods

### 2.1. Study Design

This study was a clustered, randomized, parallel, multicenter clinical trial that was approved by the Taipei Medical University (TMU)-Joint Institutional Review Board (ID: N202011065, 27 January 2021) and ClinicalTrials.gov Protocol Registration and Results System (NCT04857463, 20 April 2021).

Participants who met the criteria were divided into two groups: an NE (NE) group and an NSD group. The experimental period was 12 weeks. Participants in the NE group received NE offered by a dietitian, twice in the first 6 weeks and once in the last 6 weeks. For the NE, the Geriatric Nutrition Handbook from the Taiwan Health Promotion Administration was used, which includes daily dietary guidelines and how to eat when dysphagia and gastrointestinal discomfort occur. Except for regular nutritional education in the NE group, the NSD group was provided with two packs of NSD (Mei Balance, Meiji Holdings, Tokyo, Japan) per day as a snack between meals and before bed. The nutrient contents of the NSD are shown in Table 1. The body composition, muscle strength, nutritional status, and blood biomarkers were evaluated at the baseline, in the sixth week, and at the end of the study at 12 weeks. A survey of the quality of life (QOL) and the health status during the experimental period were monitored at the baseline and the end of the study.

### 2.2. Participant Recruitment

Regarding inclusion criteria, participants who were older than 65 years and had the Malnutrition Universal Screening Tool (MUST) score of ≥1 were considered at risk of malnutrition. The MUST score was evaluated by three factors such as BMI, unplanned weight loss, and acute disease effects. Additionally, participants were not allowed to accept any ONSs at the time of recruitment for this study. The exclusion criteria included subjects who have chronic diseases, such as diabetes undergoing insulin therapy, chronic kidney disease, and cancer. Hypertension and hyperlipidemia, without taking medicine, were not excluded in this study.

There were four nursing homes in this study, including (1) Taipei Veterans Home, Veterans Affairs Council, (2) Ren-ai Senior Citizens Home, New Taipei City Government, (3) Northern Region Senior Citizens’ Home, Ministry of the Interior, and (4) Hygge Healthcare. The group assignment was determined through a random selection process, where participants within the same nursing home were assigned to the same group. This was carried out to avoid participants comparing whether they received oral nutritional supplements with each other. As a result, (1) and (2) were NE groups, while (3) and (4) were NSD groups. Regarding the recruitment process, the staff members of the project took the opportunity to explain the details of the experiment to the participants during the group activities. This included providing information about the purpose of the study, the intervention being tested, and the potential benefits and risks. Staff members also emphasized the voluntary nature of participation and assured them of their right to withdraw from the study at any time without consequences. After the explanation, those participants who expressed a willingness to participate were asked to remain for further assessment, such as the MSUT score and medical history. Participants who achieved inclusion/exclusion criteria were then given the opportunity to discuss their participation with their family members. Finally, participants who expressed continued willingness to participate after discussing with their family members were asked to sign a consent form. This formalized their agreement to be part of the experiment and marked their official entry into the study.

### 2.3. Anthropometric Data

Anthropometric measurements were conducted using Karada scan 371 (Omron Kabushiki-Gaisha, Kyoto, Japan) including BW, percentage of muscle mass, and percentage of body fat. The appendicular skeletal muscle mass index (ASMI) was calculated based on the BW, percentage of muscle mass, and height. The calculation formula was BW (kg) × muscle mass (%) ÷ height^2^ (m) = ASMI (kg/m^2^). The normal ASMI was defined based on the Asian Working Group for Sarcopenia: 2019 consensus update on sarcopenia diagnosis and treatment. Regarding the measurement method for calf circumference, a tape measure was used to determine the circumference around the thickest part of the calf.

### 2.4. Blood Pressure

Blood pressure data, including systolic (SBP) and diastolic blood pressure (DBP), were collected by daily records of the nursing home and were measured with an Omron HBP-9020 (Omron Corporation, Tokyo, Japan).

### 2.5. Physical Performance

The Study of Osteoporotic Fractures (SOF) index was used to assess the frailty of older participants [20]. Hand grip strength was measured with a Camry eh101 hand dynamometer (Camry Scale, South El Monte, CA, USA). Walking speed was determined by a 6-m walking test [21].

### 2.6. Dietary Calorie Intake and Nutritional Status

The daily calorie intake and the Mini-Nutritional Assessment Short Form (MNA-SF) were assessed by a dietitian. Regarding the dietary intake, since the nursing home provided the same meals to all residents; the calorie intake was estimated based on the remaining portion of each participant’s meal. The MNA-SF was used to assess the nutritional status, with scores of 12~14 considered no risk of malnutrition, 8~11 considered at risk of malnutrition, and 0~7 considered malnutrition [22].

### 2.7. Blood Biochemical Analyses

Fasting blood samples were analyzed with the ADVIA 1800^®^ Clinical Chemistry System (Siemens Healthcare, Erlangen, Germany), including blood cell analysis, liver function indicators (aspartate transaminase, AST; alanine transaminase, ALT), blood sugar, blood triglycerides, a nutritional status indicator (albumin level), and renal function indicators (uric acid and creatinine levels). A total of 25(OH) vitamin D (Vit. D) was analyzed by a chemiluminescence immunoassay using Abbott Alinity I (Abbott Park, IL, USA). Serum zinc was analyzed by NexION 350 Series (PerkinElmer, Waltham, MA, USA).

### 2.8. QOL and Health Status

The physical and mental components scale of the MOS 36-Item Short Form Health Survey (SF-36) were used to monitor QOL and the health status [23,24].

### 2.9. Statistical Analysis

All values are expressed as the mean and standard deviation (SD) or *n* and percentage (%). SPSS vers. 22 (IBM, Armonk, NY, USA) was used to evaluate differences between groups and changes in parameters. The Shapiro-Wilk test was used to determine the normality. Parametric data were compared with a paired *t*-test, *t*-test, or Wilcoxon signed-rank test, and the Mann-Whitney U test was used to analyze non-parametric data. Frequencies between groups were compared by Fisher’s exact test or a Chi-squared test. A multiple linear regression was used to evaluate parameters associated with the intervention. A logistic regression was used to determine the odds ratio of parameters. Pearson’s correlation or Spearman’s rank correlation was used to examine correlations between the albumin level and different parameters. The power of the current study was 0.99 as calculated by G*Power software vers. 3.1 (Heinrich-Heine-Universität Düsseldorf, Düsseldorf, Germany) based on the mean of the MNA-SF at the 12th week and setting α to 0.05. Statistical significance was accepted at *p* < 0.05.

## 3. Results

### 3.1. Basic Information

**Figure 1:**
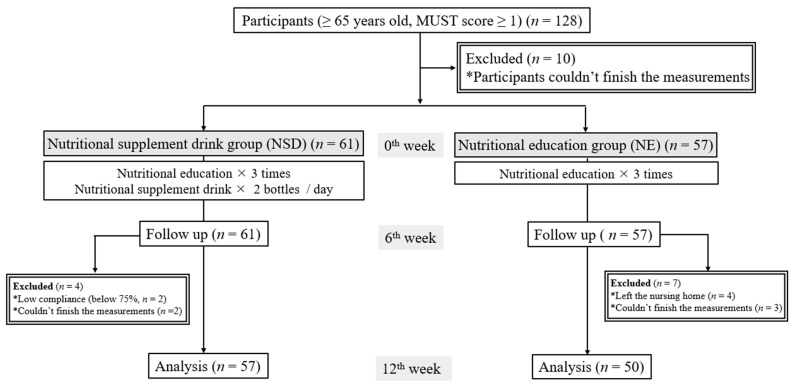
Study flow diagram for nutritional supplement drink or nutritional education on the nutritional status in older individuals at risk of malnutrition. MUST, Malnutrition Universal Screening Tool.

A study flow diagram is shown in Figure 1. Recruitment for this study began in November 2021 and ended in March 2023. In total, 128 participants met the inclusion criteria; however, 10 participants could not finish the measurements. Sixty-one participants entered the NSD group, while 57 participants entered the NE group. After 6 weeks of the intervention, four participants in the NSD group dropped out due to low compliance and being unable to finish the measurements. Seven participants dropped out in the NE group at the follow-up time point in the 6th week for leaving the nursing home (*n* = 4) or being unable to finish the measurements (*n* = 3). After 12 weeks of the intervention, 57 participants completed the study in the NSD group, and 50 participants in the NE group completed the study. Compliance was 75% based on the number of empty bottles collected from participants.

### 3.2. Baseline Demographics, Anthropometric Assessments, and Dietary Intake of Study Participants

As shown in Table 2, participants in the NE group were significantly younger than those in the NSD group. At the baseline, the NE group had significantly higher BW, BMI, and ASMI, compared to the NSD group. On the other hand, the numbers, or percentages of each MUST score significantly differed between the two groups at the baseline. The NSD group had more participants whose MUST score was 2 or 3.

### 3.3. Body Composition, Physical Performance, and Nutritional Status

#### 3.3.1. Changes after the NSD Intervention

In the NSD group, compared to the baseline, BW and BMI had significantly increased after 12 weeks of the NSD intervention. The SOF score had significantly decreased while walking speed had significantly increased at the endpoint compared to the baseline. Moreover, the MNA-SF score was significantly higher at the endpoint compared to the baseline (Table 3).

#### 3.3.2. Changes after the NE Intervention

In the NE group, there were no differences in any parameters after 12 weeks of NE (Table 3).

#### 3.3.3. Comparisons of the Effects between NSD and NE

Table 4 displays the changes in anthropometric data and nutritional status, considering the differences in baseline BW, BMI, and ASMI between the two groups. Compared to the NE group, the elevation of BW and BMI was significantly higher in the NSD group. Moreover, the SOF score significantly decreased, whereas the walking speed was significantly higher in the NSD group compared to the NE group. The NSD group had significantly increased caloric intake compared to the NE group. In addition, based on the MNA-SF score, the NSD group showed obvious improvement in the nutritional status compared to the NE group.

### 3.4. Blood Biochemical Analysis

Blood biochemical parameters are shown in Supplementary Table S2. Although values of several items undulated, they were still in an acceptable range, such as white blood cell count (WBC), mean corpuscular hemoglobin (MCH), mean corpuscular hemoglobin concentration (MCHC), percentages of neutrophils and lymphocytes, AST activity, ALT activity, and albumin level in the NSD group as well as MCH, mean corpuscular volume (MCV), and triglyceride levels in the NE group. Blood Vit. D and zinc levels are discussed further in the next paragraph. The blood albumin level significantly decreased in the NSD group after 12 weeks of the intervention. Albumin levels were 4.28 ± 0.27, 4.18 ± 0.32, and 4.19 ± 0.32 g/dL in the NSD group, and 4.17 ± 0.41, 4.21 ± 0.42, and 4.15 ± 0.47 g/dL in the NE group, at weeks 0, 6, and 12, respectively. Changes in albumin level (week 12—the baseline) were −0.09 ± 0.28 g/dL in the NSD group and −0.02 ± 0.34 g/dL in the NE group (NSD vs. NE *p* = 0.252). However, albumin levels were still within a normal range in both the NSD and NE groups.

### 3.5. Vit. D and Zinc Deficiencies

#### 3.5.1. Changes after the NSD Intervention

In the NSD group, 42.1% of participants exhibited a Vit. D deficiency and 31.6% of participants had an insufficient level at the baseline (Table 5). After the 12-week intervention, there was no change in the Vit. D status. As to the zinc status, 49.1% of participants had a deficient level at the beginning of the experiment (Table 5). However, no change was found in the zinc status after 12 weeks.

#### 3.5.2. Changes after the NE Intervention

In the NE group, 42.0% of participants had a deficiency and 42.0% of participants had an insufficient level of Vit. D at the baseline (Table 5). After 12 weeks, no change was found in the Vit. D status. As to the zinc status, 24.0% of participants exhibited a zinc deficiency at the baseline. However, the percentage of participants with a zinc deficiency had significantly increased after 12 weeks (Table 5).

#### 3.5.3. Comparison of the Vit. D and Zinc Statuses between the NSD and NE Groups

The NE group had more participants with sufficient zinc at the baseline compared to the NSD group. However, there were no differences in percentages of participants with deficiencies in the Vit D and zinc statuses at the end of the study between the two groups (Table 5).

### 3.6. Effects of NSDs on Variables at Week 12, as Analyzed by a Multiple Linear Regression

#### 3.6.1. Body Composition, Physical Performance, and Nutritional Status

As shown in Table 6, BW, BMI, calf circumference, walking speed, and the MNA-SF score were positively significantly affected by the NSD, while the SOF score was negatively significantly affected by the NSD (Table 6). Results indicated that the NSD could increase the BW, BMI, calf circumference, walking speed, and MNA-SF score, and improve frailty.

#### 3.6.2. Blood Biochemical Analysis

Table 7 shows biochemical parameters analyzed by the multiple linear regression. The albumin level was positively affected by the NSD, while the eosinophil percentage was negatively affected by the NSD (Table 7).

### 3.7. Odds Ratio (OR)

Crude ORs are shown in Table 8, and the risks of frailty, malnutrition, and low walking speed were significantly reduced after the NSD intervention. After the NSD intervention, models 1 and 2 showed the same trend of the risks of frailty, malnutrition, and low walking speed being reduced.

However, the risk of a low ASMI increased after the NSD intervention in models 1 and 2. After adjusting for the baseline ASMI and other parameters in model 2, the risk of a low ASMI disappeared in model 3. Therefore, it was estimated that the higher risk of a low ASMI might be related to the significantly lower ASMI in the NSD group at the baseline.

In model 3, the NSD intervention produced a lower risk of zinc deficiency.

### 3.8. QOL and SF-36 Questionnaire

#### 3.8.1. Effects of the NSD or NE Interventions

After 12 weeks of the intervention, the NSD group exhibited significant improvements in physical function (PF), vitality (VT), mental health (MH), and also the total score of physical health (physical component score, PCS) and total mental health (mental component score, MCS) in the SF-36 questionnaire (Supplementary Table S3). Results indicated that the NSD intervention improved the vitality and mental health of nursing home-dwelling older persons. There were no differences between the endpoint and the baseline for each item of the SF-36 questionnaire except for PF in the NE group, as PF had significantly decreased at the endpoint compared to the baseline (Supplementary Table S3).

#### 3.8.2. Changes in SF-36 Questionnaire Scores after the NSD and NE Interventions

To evaluate changes and improvements in SF-36 scores after the intervention, we compared changes in scores of both groups by analyzing values of week 12 minus those of week 0. The NSD group had significantly greater improvements in PF, VT, social functioning (SF), MH, PCS, and MCS than the NE group (Supplementary Table S4).

### 3.9. Correlations among the SF-36 Questionnaire, the Nutritional Status, Physical Performance, Vit. D, and the Intervention

As shown in Supplementary Table S5, after the 12-week intervention, PF, general health (GH), VT, and MH were positively correlated with the MNA-SF. PF, GH, and PCS were positively correlated with walking speed, while GH was positively correlated with the albumin level. Moreover, the NSD intervention was positively correlated with PF, GH, VT, MH, and MCS. Based on these results, a better nutritional status was associated with a higher SF-36 score, which included PF and MH.

## 4. Discussion

### 4.1. ONSs, BW, Body Composition, and Physical Performance

The current results demonstrated that NSDs significantly increased BW and BMI (Table 3, NSD group). One report indicated that the excess energy requirement for 1 kg of weight gain in young women with anorexia nervosa was approximately 7500 kcal/kg BW [25]. In contrast, the excess energy requirement was shown to be 8856 to 22,620 kcal/kg BW in malnourished nursing home patients [26]. Therefore, it was suggested that amelioration of a malnourished state is more difficult in the older than in young individuals [27]. In this study, BW gain was 1.21 ± 1.83 kg after 3 months of the NSD intervention (Table 4, NSD group). The excess total energy intake was around 35,813 (397.92 × 90 days) kcal (Table 4, energy intake). Therefore, it was speculated that the NSD provided more calories and nutrients which contributed to elevated BW and BMI. In addition, according to the Dietary Reference Intake (DRI) values in Taiwan, the recommended daily energy intake of older adults (older than 65 years) is 1900 kcal for males and 1500 for females. In this study, energy intake was insufficient at the baseline (Table 3). This is an important issue for increasing the energy intake of older nursing home residents. Detailed nutrient intake levels should be evaluated in future studies.

In this study, the SOF significantly decreased, and walking speed significantly increased without changing the body fat or muscle mass in the NSD group (Table 3). In this study, a cafeteria diet was provided to nursing home residents except during the period of coronavirus disease 2019 (COVID-19) restrictions. Interestingly, outdoor activities were also restricted during the study period because of COVID-19 transmission prevention, even though the SOF and walking speed improved in the NSD group. Loman et al. indicated that ONS consumption increased the intake of numerous nutrients without decreasing nutrient intake from food in older malnourished adults post-discharge [28]. Anton et al. also reported that dietary interventions had inconsistent effects on functional and strength outcomes; however, exercise interventions and combined diet and exercise interventions consistently improved lower-body muscle strength but had less consistent effects on walking speed and grip strength [29]. Therefore, in this study, it is undeniable that the NSDs contributed to decreasing frailty based on the SOF and the increase in the walking speed in older residents. However, physical activities should be monitored in future studies.

Compared to participants who only received the NE intervention, the improved effects of the NSD intervention on increasing BW, BMI, and ASMI and decreasing the SOF and physical performance were more obvious than those of the NE intervention (Table 4). As a result, providing ONS can be effective in improving the nutritional status of older nursing home residents.

### 4.2. Effects of the NSD on Related Variables

Based on a multiple linear regression, NSDs showed positive effects on BW, BMI, calf circumference, walking speed, and MNA-SF scores, and negative effects on the SOF, and produced an improvement in frailty (Table 6). Phillips et al. indicated that the MNA-SF and MUST are practical nutritional screening tools for community-dwelling seniors [30]. In Japan, Nakamura et al. discovered that the MNA had the highest area under the curve (AUC) score for age-related muscle loss and sarcopenia when using the criteria of the Asian Working Group for Sarcopenia (AWGS) [31]. Poulia et al. also noted that the MNA-SF and MUST tools are practical, as they can be used in clinical settings and all community areas [32]. In this study, MUST was used to evaluate the risk of malnutrition because the information could be checked based on the health records of the nursing homes. At the baseline, both groups showed a risk of malnutrition based on MUST as well as the MNA-SF (Table 3). After 12 weeks, the NSD group had MNA-SF scores of >12, which represented a normal nutritional status [22].

### 4.3. Blood Albumin Levels and Related Variables

Blood albumin levels remained within the normal range in both the NSD and NE groups, although it had significantly decreased in the NSD group after 3 months (Supplementary Table S2). As shown in Supplementary Table S6, calf circumference, ASMI, grip strength, walking speed, and MNA-SF scores were positively correlated with the blood albumin level, but without strong correlations. According to previous studies, albumin levels decrease with aging, and older adults exhibit greater decreases in albumin [33,34], which was also observed in this study. The NSD group had a higher average age, which may explain why decreases in the albumin level were more apparent in the NSD group than in the NE group. In addition, a meta-analysis by Cabrerizo et al. and a cohort study by Kitamura et al. showed that albumin levels were associated with physical activities, activities of daily living (ADLs), and muscle mass, which is related to inadequate protein intake [34,35]. Therefore, nutrient intake, especially protein, and physical activities should be monitored in future studies.

### 4.4. Vit. D and Zn Deficiencies

In this study, the definition of the Vit. D status was based on recommendations from the Endocrine Society Task Force on Vitamin D in the US population [36]. It was found that 78.5% (84/107) of participants exhibited Vit. D insufficiency or deficiency (Table 5). In Europe, the prevalence of Vit. D deficiency in the general population is as high as 37% and as high as 80% in nursing home residents and non-European immigrants [37]. A study in a Swedish community involving 22 nursing homes also indicated a significant number of older persons with a low Vit. D condition with a mean of 34 ng/mL, and 82% had a value of <20 ng/mL [38]. In Taiwan, a study among community-based older persons found most of the deficiency in Vit. D was 22.4% as serum 25(OH)D which was <20 ng/mL, and the mean level of participants was <30 ng/mL [39]. Unfortunately, there is limited study of awareness among nursing home dwellers in Taiwan to evaluate the prevalence of Vit. D deficiency. After the 12-week NSD intervention, the Vit. D status had not improved in participants, which might be because the drink was not a Vit. D-fortified product (Table 1 and Table 8). The Vit. D status of older adults must be highlighted in nutritional care.

The percentage of participants with a Zn deficiency was 37.3% (40/107) at the baseline in this study (Table 5). In addition, the percentage with a Zn deficiency did not change in the NSD group, while it significantly increased in the NE group after the 12-week intervention. Zinc is among the minerals whose consumption or bioavailability may drop in older populations, as influenced by reduced absorption, an increasing number of diseases that alter the use of zinc, and increased use of drugs that reduce its bioavailability in the body [40]. IZiNCG recommends that if more than 20% of a population’s serum Zn concentrations are below a critical value, the population’s risk of a Zn deficiency is considered to have increased [41]. A limited amount of research has been conducted on the Zn status of older adults. As a result, the ZN status may soon become an important health issue.

### 4.5. Crude and Adjusted ORs

The crude and adjusted ORs showed risks of frailty, malnutrition, and low walking speed were significantly reduced after the NSD intervention (Table 8). However, the risk of low ASMI increased in models 1 and 2, whereas there was no change in model 3 after the NSD intervention (Table 8). An ASMI evaluation can contribute important information to an assessment of the nutritional status because it reflects the body’s protein mass [42]. Gallagher et al. also demonstrated that skeletal muscles are affected by age, gender, and ethnicity [43]. Based on Table 8, ORs for frailty, malnutrition, and a low walking speed were significantly reduced, whereas the OR of low ASMI was significantly elevated after the NSD intervention. Nevertheless, after adjusting for age, sex, baseline BMI, and ASMI, the OR for low ASMI became nonsignificant. Possible reasons might be because of the significant difference in age, BMI, or ASMI between the two groups and the measurement tools of muscle mass. Common methods to determine the appendicular skeletal muscle mass (ASMM) are a bioelectrical impedance analysis (BIA) and dual-energy X-ray absorptiometry (DEXA) [44]. However, Kuriyan also pointed out that several factors must be considered when selecting an appropriate method, including the feasibility, cost, technical skills required, level of accuracy, participant burden, radiation exposure, the time required, validation in an appropriate population, and the availability of reference data [45]. Therefore, a portable electronic body composition scale was utilized in this study, since it is easy to move between different areas and is less burdensome for older individuals.

### 4.6. Nutritional Status and QoL

Results indicated that NSDs can improve the activity and mental health of older nursing home-dwelling persons more than those who only received NE (Supplementary Tables S3 and S4). In a previous cohort study of older men living in veteran homes, it was found that the non-malnourished population had a higher percentage of better ADLs and better moods than the malnourished population [46]. Results of another study, which recruited 72 participants with an average age of 70.8 years in two nursing homes, indicated that higher MNA scores were associated with a lower tendency for depression and better physical functioning and ADLs. It was proposed that older persons with better physical performance may be better equipped to handle everyday activities associated with eating and physical activities [47]. Similarly, the intervention with a nutritional supplement had positive correlations with both physical function and mental health in this study.

### 4.7. Strengths and Limitations

In this study, the statistical power was 0.99, which indicates a high possibility of identifying a true effect. Additionally, several nursing homes participated in the study. However, some limitations of this study should be addressed. First, the study was conducted between November 2021 and March 2023, when Taiwan was affected by COVID-19. To prevent the spread of COVID-19, nursing homes had strict requirements, including controlling visitor entry, weekly rapid tests on residents and caregivers, and quarantine measures. This policy and situation both limited researchers from collecting the questionnaires face-to-face with residents and may have created biases due to self-reporting of the questionnaires. Second, since the dietitian only provided a summary of total energy intake, nutrient intake details were not calculated. Third, a portable body composition scale was used in this study because it can conveniently be moved between nursing homes. Although the portable body composition scale was cited in previous studies [8,48,49], it should be replaced in the future by more reliable measurements such as BIA or DEXA. Lastly, apart from Vit. D and zinc, other common nutrients that are prone to deficiency in older adults, such as Ca, iron, Vit. B_12_, folic acids, etc. Therefore, it is necessary to clarify these kinds of nutrients in future studies.

## 5. Conclusions

**Figure 2:**
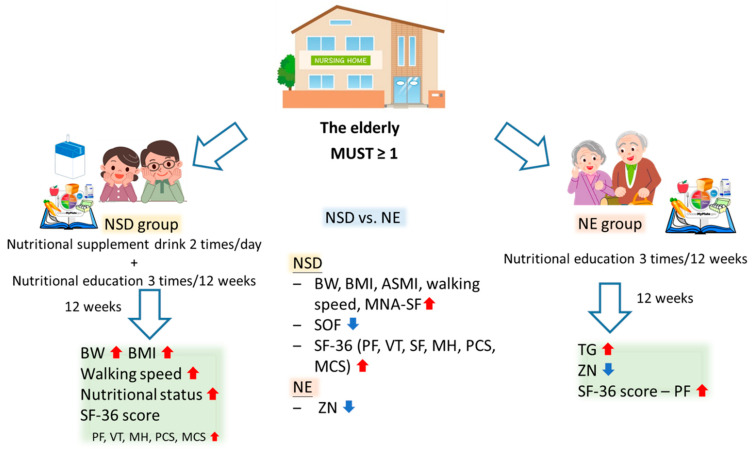
Beneficial effects of a nutritional supplement drink (NSD) on the nutritional status of older individuals at high risk of malnutrition. The upward arrow represents an increase or growth, while the downward arrow represents a decrease or decline. NE, nutritional education; MUST, malnutrition universal screening tool; BW, body weight; BMI, body-mass index; ASMI, appendicular skeletal muscle index; SOF, study of osteoporotic fractures; MNA-SF, Mini-Nutritional Assessment Short Form; TGs, triglycerides; SF-36, MOS 36-Item Short Form Health Survey; PF, physical functioning; VT, vitality; MH, role emotional; PCS, physical component score; MCS, mental component score.

Current results indicated that the NSD intervention combined with NE for 12 weeks improved the nutritional status, including BW, BMI, and MNA-SF scores, as well as physical performance such as walking speed in the older population at risk of malnutrition (Figure 2). Moreover, SF-36 questionnaire scores also increased, including the physical and mental health scales. In conclusion, ONS was effective in improving the nutritional status of older nursing home residents who were at risk of malnutrition.

## Figures and Tables

**Table 1:** Nutrition Facts of Meiji Mei Balance *.

125 mL per Pack					
Energy		203 kcal			
		1.62 kcal/mL	Vitamin B_12_		1.2 μg
Protein		7.5 g	Folic acid		60 mg
Branched-chain amino acids ^1^		1580 mg	Pantothenic acid		1.2 mg
Leucine		720mg	Biotin		30 μg
Fat		5.6 g	Vitamin C		100 mg
Saturated fat		1.2 g	Choline		1.5 mg
Trans-fat		0 g	Potassium	(sweet flavor ^2^)	180 mg
Carbohydrates		31.8 g		(corn soup flavor)	200 mg
Sugar		5.8 g	Chlorine	(sweet flavor ^2^)	120 mg
Dietary fiber		2.5 g		(corn soup flavor)	220 mg
Sodium	(sweet flavor ^2^)	130 mg	Calcium		120 mg
	(corn soup flavor)	180 mg	Phosphorus		140 mg
Vitamin A		120 μg RE	Magnesium		40 mg
Vitamin D		1.2 μg	Iron		1.5 mg
Vitamin E		6 mg α-TE	Zinc		2 mg
Vitamin K		19 μg	Manganese		0.46 mg
Vitamin B_1_		0.3 mg	Iodine		30 μg
Vitamin B_2_		0.4 mg	Selenium		12 μg
Niacin		5.4 mg NE	Molybdenum		10 μg
Vitamin B_6_		0.6 mg	Chromium		6 μg

* Mei Balance (Meiji Holdings, Tokyo, Japan). ^1^ Branched-chain amino acids included valine, leucine, and isoleucine. ^2^ Sweet flavors included strawberry, banana, yogurt, and coffee.

**Table 2:** Baseline demographic information, anthropometric assessment results, and dietary intake levels of study participants.

	NSD (*N* = 57)	NE (*N* = 50)	*p*-Value
Age (years)	80.67 ± 9.03	75.86 ± 8.21	0.007
<75, *n* (%)	38 (66.7)	24 (48.0)	0.051
≥75, *n* (%)	19 (33.3)	26 (52.0)	
Sex			
Male *n*, (%)	46 (80.7)	32 (64.0)	0.052
Female *n*, (%)	11 (19.3)	18 (36.0)	
Height (cm)	162.04 ± 7.61	162.47 ± 9.39	0.793
BW (kg)	53.73 ± 8.31	62.91 ± 13.57	<0.001
BMI (kg/m^2^)	20.47 ± 3.03	23.74 ± 4.30	<0.001
Body fat (%)	25.97 ± 5.92	27.03 ± 5.08	0.326
ASMI (kg/m^2^)	5.38 ± 0.95	6.38 ± 1.38	<0.001
Normal, *n* (%)	5 (8.8)	24 (48.0)	<0.001
Low, *n* (%)	52 (91.2)	26 (52.0)	
Calf circumference (cm)	31.98 ± 3.40	32.95 ± 4.05	0.177
Normal, *n* (%)	20 (35.1)	25 (50.0)	0.169
Low, *n* (%)	37 (64.9)	25 (50.0)	
SBP (mmHg)	133 ± 17	132 ± 17	0.871
DBP (mmHg)	78 ± 11	77 ± 10	0.609
MUST			
1, *n* (%)	37 (64.9)	41 (82.0)	0.007
2, *n* (%)	19 (17.8)	9 (18.0)	
3, *n* (%)	10 (9.3)	0 (0.0)	
MNA-SF	9.07 ± 1.83	10.66 ± 1.92	<0.001
Normal 12–14, *n* (%)	7 (12.3)	20 (40.0)	0.002
Low 0–11, *n* (%)	50 (87.7)	30 (60.0)	
Total energy intake (kcal)	1213.86 ± 69.53	1219.00 ± 67.68	0.315
SOF	0.98 ± 0.64	0.50 ± 0.61	<0.001
Normal, *n* (%)	12 (21.1)	28 (56.0)	<0.001
pre-frailty & frailty, *n* (%)	45 (78.9)	22 (44.0)	
Grip strength (kg)	21.66 ± 6.14	21.64 ± 8.09	0.991
Normal, *n* (%)	13 (22.8)	16 (32.0)	0.384
Low, *n* (%)	44 (77.2)	34 (68.0)	
Walking speed (m/s)	0.73 ± 0.30	0.83 ± 0.20	0.089
Normal, *n* (%)	13 (21.1)	8 (16.0)	0.467
Slow, *n* (%)	44 (77.2)	42 (84.0)	
Hypertension, *n* (%)	47 (82.0)	41 (82.0)	1.000
Hypercholesterolemia, *n* (%)	6 (10.5)	12 (24.0)	0.074
Hyperglyceridemia, *n* (%)	7 (12.3)	8 (16.0)	0.591

Data are expressed as the mean ± standard deviation. The Shapiro-Wilk test was used to determine the normality of the population. Data were compared by a *t*-test or Mann-Whitney U test. Frequencies between groups were compared by Fisher’s exact test or a Chi-squared test. Normal ASMI in males ≥ 7.0 kg/m^2^, females ≥ 5.7 kg/m^2^; MNA-SF 12–14 was considered as well-nourished; SOF 1 was considered as pre-frailty, ≥2 was considered as frailty; normal grip strength in male ≥ 28 kg, female ≥ 18 kg; normal walking speed ≥ 1.0m/s; calf circumference > 34 cm in male > 33 cm in female was considered normal; hypertension is defined as SBP > 120 mmHg, DBP > 80 mmHg; hypercholesterolemia is defined as blood total cholesterol level > 200 mg/dL; hyperglyceridemia is defined as blood triglyceride level > 150 mg/dL. NSD, nutritional supplement drink; NE, nutritional education; BW, body weight; BMI, body-mass index; ASMI, appendicular skeletal muscle index; SBP, systolic blood pressure; DBP, diastolic blood pressure.

**Table 3:** Effects of a nutritional supplement drink (NSD) on the body composition, physical performance, nutritional status, and nutrition intake in older nursing home residents at risk of malnutrition.

	NSD (*N* = 57)			NE (*N* = 50)		
	*p*-Value			*p*-Value		
	Baseline	End	Baseline × End	Baseline	End	Baseline × End
Body composition						
BW (kg)	53.73 ± 8.31	54.94 ± 8.50	<0.001	62.91 ± 13.57	62.73 ± 12.67	0.574
BMI (kg/m^2^)	20.47 ± 3.03	20.97 ± 3.03	<0.001	23.74 ± 4.30	23.67 ± 3.98	0.603
Body fat (%)	25.97 ± 5.92	26.58 ± 6.13	0.273	27.03 ± 5.08	27.43 ± 4.16	0.356
Muscle mass (%)	26.31 ± 3.03	26.15 ± 3.13	0.499	26.81 ± 2.81	26.49 ± 2.92	0.313
ASMI (kg/m^2^)	5.38 ± 0.95	5.46 ± 0.97	0.189	6.38 ± 1.38	6.30 ± 1.33	0.263
Calf circumference (cm)	31.98 ± 3.40	32.14 ± 3.01	0.842	32.95 ± 4.05	32.57 ± 3.91	0.065
Physical performance						
SOF	0.98 ± 0.64	0.23 ± 0.42	<0.001	0.50 ± 0.61	0.56 ± 0.58	0.180
Grip strength (kg)	21.66 ± 6.14	21.97 ± 6.21	0.481	21.64 ± 8.09	21.88 ± 7.74	0.621
6-m walking speed (s)	10.02 ± 5.04	8.56 ± 4.79	0.001	7.71 ± 2.12	8.16 ± 2.89	0.111
Walking speed (m/s)	0.73 ± 0.30	0.89 ± 0.38	<0.001	0.83 ± 0.20	0.83 ± 0.32	0.363
Blood pressure						
SBP (mmHg)	133 ± 17	133 ± 15	0.857	132 ± 17	131 ± 14	0.251
DBP (mmHg)	78 ± 11	76 ± 8	0.079	77 ± 10	76 ± 9	0.574
Nutritional status						
MUST						
0, *n* (%)	0 (0.0)	34 (59.6)	<0.001	0 (0.0)	2 (3.8)	0.355
1, *n* (%)	37 (64.9)	12 (21.1)		41 (78.8)	40 (76.9)	
2, *n* (%)	10 (17.5)	9 (15.8)		9 (17.3)	8 (15.4)	
3, *n* (%)	10 (17.5)	2 (3.5)		0 (0.0)	0 (0.0)	
MNA-SF	9.07 ± 1.83	12.04 ± 1.31	<0.001	10.66 ± 1.92	10.58 ± 1.93	0.773
Total energy intake (kcal)	1213.9 ± 69.53	1611.8 ± 71.21	<0.001	1219.0 ± 67.68	1223.0 ± 59.08	0.417

Data are expressed as the mean ± standard deviation. The Shapiro-Wilk test was used to determine the normality of the population, and data were compared by a *t*-test, Mann-Whitney U test, paired *t*-test, Wilcoxon signed-rank test, or Chi-squared test. NE, nutritional education; BW, body weight; BMI, body-mass index; ASMI, appendicular skeletal muscle index; SOF, the study of osteoporotic fractures; MUST, malnutrition universal screening tool; MNA-SF, Mini-Nutritional Assessment Short Form.

**Table 4:** Changes in anthropometric data and the nutritional status after 12 weeks of the intervention.

	NSD (*N* = 57)	NE (*N* = 50)	NSD × NE
	Week12-Baseline	Week12-Baseline	*p*-Value
Body composition			
BW (kg)	1.21 ± 1.83	−0.17 ± 2.17	<0.001
BMI (kg/m^2^)	0.5 ± 0.78	−0.07 ± 0.88	<0.001
Body fat (%)	0.62 ± 4.21	0.40 ± 3.06	0.783
Muscle mass (%)	−0.16 ± 1.83	−0.33 ± 2.26	0.685
ASMI (kg/m^2^)	0.09 ± 0.48	−0.08 ± 0.54	0.016
Calf circumference (cm)	0.17 ± 2.77	−0.39 ± 1.46	0.191
Physical performance			
SOF	−0.75 ± 0.69	0.06 ± 0.31	<0.001
Grip strength (kg)	0.31 ± 3.34	0.19 ± 2.02	0.428
6-m walking speed (s)	−1.47 ± 3.23	0.45 ± 2.12	0.001
Walking speed (m/s)	0.15 ± 0.24	0.00 ± 0.29	0.004
Nutritional status			
Total energy intake (kcal)	397.92 ± 29.90	4.00 ± 31.68	<0.001
MNA-SF	2.96 ± 1.86	−0.08 ± 1.28	<0.001

Data were calculated by the value of week 12—the baseline and are expressed as the mean ± standard deviation. The Shapiro-Wilk test was used to determine the normality of the population. Data were compared by a *t*-test or Mann-Whitney U test. NSD, nutritional supplement drink; NE, nutritional education; BW, body weight; BMI, body mass index; ASMI, appendicular skeletal muscle index; SOF, study of osteoporotic fractures; MUST, malnutrition universal screening tool; MNA-SF, Mini-Nutritional Assessment Short Form.

**Table 5:** Effects of nutritional supplement drink (NSD) on the plasma vitamin (Vit.) D and zinc status in older nursing home residents at risk of malnutrition.

	NSD			NE			NSD × NE	
	*p*-Value			*p*-Value			*p*-Value	
	Baseline	End	Baseline × End	Baseline	End	Baseline × End	Baseline	End
25-OH Vit. D (ng/mL)								
Deficiency (≤20 ng/mL), *n* (%)	24 (42.1)	23 (40.4)	0.923	21 (42.0)	21 (42.0)	0.956	0.348	0.355
Insufficiency (21~29.9 ng/mL), *n* (%)	18 (31.6)	20 (35.1)		21 (42.0)	22(44.0)			
Sufficiency (≥30 ng/mL), *n* (%)	15 (26.3)	14 (24.6)		8 (16.0)	7 (14.0)			
Zinc (μg/L)								
Deficiency (<700 μg/L), *n* (%)	28 (49.1)	23 (40.4)	0.451	12 (24.0)	23 (46.0)	0.035	0.009	0.565
Sufficiency (≥700 μg/L), *n* (%)	29 (50.9)	34 (59.6)		38 (76.0)	27 (54.0)			

Data are expressed as numbers and percentages. Frequencies between groups were compared by Fisher’s exact test or a Chi-squared test. NE, nutritional education. 25-OH Vit.D, 25-Hydroxyvitamin D.

**Table 6:** Effects of a nutritional supplement drink on the body composition, physical performance, nutritional status, and nutrition intake of older nursing home residents at risk of malnutrition by multiple linear regression.

12th Week	β	*p*-Value	*R* ^2^
Body composition			
BW (kg)	0.041	0.030	0.971
BMI (kg/m^2^)	0.048	0.040	0.956
Body fat (%)	0.067	0.511	0.135
Muscle mass (%)	0.008	0.938	0.191
ASMI (kg/m^2^)	0.020	0.722	0.726
Calf circumference (cm)	0.253	0.002	0.476
Physical performance			
SOF	−0.326	0.002	0.080
Grip strength (kg)	0.160	0.090	0.258
Walking speed (m/s)	0.094	0.026	0.094
Blood pressure			
SBP (mmHg)	0.034	0.755	0.003
DBP (mmHg)	−0.079	0.472	−0.003
Nutritional status			
MNA-SF	0.705	<0.001	0.512

Data were analyzed by multiple linear regression and were adjusted for age, baseline BW, and baseline BMI. BW, body weight; BMI, body mass index; ASMI, appendicular skeletal muscle index; SOF, study of osteoporotic fractures; SBP, systolic blood pressure; DBP, diastolic blood pressure; MNA-SF, Mini-Nutritional Assessment Short Form.

**Table 7:** Effects of a nutritional supplement drink on biochemical parameters in older nursing home residents at risk of malnutrition by multiple linear regression.

12th Week	β	*p*-Value	*R* ^2^
Blood sugar (AC) (mg/dL)	0.181	0.093	0.043
Lipid profile			
Cholesterol (mg/dL)	0.041	0.698	0.055
Triglycerides (mg/dL)	0.034	0.741	0.104
Kidney function			
Uric acid (mg/dL)	−0.048	0.653	0.027
Creatinine (mg/dL)	−0.157	0.154	−0.014
Liver function			
AST (U/L)	0.056	0.615	−0.024
ALT (U/L)	0.175	0.103	0.039
Nutritional status			
Albumin (g/dL)	0.247	0.014	0.178
Vitamin (Vit.) D status			
Total 25-OH Vit. D (ng/mL)	0.159	0.140	0.035
Zinc status			
Zinc (μg/L)	0.165	0.165	0.016
Hematology			
RBCs (10^6^/μL)	0.097	0.321	0.199
WBCs (10^3^/μL)	0.148	0.180	−0.011
Hemoglobin (g/dL)	0.166	0.087	0.222
Hematocrit (%)	0.136	0.159	0.222
Platelets (10^3^/μL)	0.084	0.434	0.041
MCH (pg)	0.098	0.376	−0.027
MCHC (g/dL)	0.186	0.087	0.020
MCV (fL)	−0.025	0.698	−0.025
Neutrophil Seg. (%)	0.072	0.517	−0.022
Lymphocytes (%)	−0.023	0.836	−0.006
Monocytes (%)	−0.011	0.955	−0.011
Eosinophils (%)	−0.250	0.022	0.030
Basophils (%)	0.002	0.985	−0.014
RDW-CV (%)	−0.006	0.957	0.015

Data were analyzed by a multiple linear regression and were adjusted for age, baseline body weight, and baseline body-mass index. ALT, alanine aminotransferase; AST, aspartate aminotransferase; MCH, mean corpuscular hemoglobin; MCHC, mean corpuscular hemoglobin concentration; MCV, mean corpuscular volume; RBCs, red blood cells; TC, total cholesterol; TIBC, total iron-binding capacity; WBCs, white blood cells; RDW-CV: red blood cell distribution width.

**Table 8:** Crude and adjusted odd ratios (ORs) (95% confidence intervals (CIs)) of variables related to oral nutritional supplement drink administration in older nursing home residents at risk of malnutrition in the 12th week.

	Crude		Model 1		Model 2		Model 3	
			Adjusted		Adjusted		Adjusted	
	OR	95% CI	OR	95% CI	OR	95% CI	OR	95% CI
Total 25-OH Vitamin D (ng/mL)								
Sufficiency (≥30 ng/mL)	1		1		1		1	
Insufficiency (20.1~29.9 ng/mL)	0.455	(0.153, 1.353)	0.416	(0.132, 1.315)	0.374	(0.108, 1.299)	0.316	(0.085, 1.174)
Deficiency (≤20 ng/mL)	0.548	(0.185, 1.618)	0.446	(0.141, 1.409)	0.373	(0.108, 1.284)	0.369	(0.101, 1.346)
Zinc (μg/L)								
Sufficiency (≥700 μg/L)	1		1		1		1	
Deficiency (<700 μg/L)	0.794	(0.369, 1.711)	0.587	(0.248, 1.391)	0.429	(0.165, 1.117)	0.356	(0.128, 0.990)
SOF								
Score = 0	1		1		1		1	
Score ≥ 1	0.273	(0.119, 0.626)	0.301	(0.126, 0.721)	0.299	(0.086, 0.612)	0.192	(0.067, 0.551)
MNA-SF								
Score 12~14	1		1		1		1	
Score 6~11	0.381	(0.174, 0.833)	0.301	(0.126, 0.721)	0.076	(0.020, 0.282)	0.058	(0.014, 0.250)
Calf circumference (cm)								
Normal	1		1		1		1	
Low calf circumference	1.704	(0.779, 3.727)	2.213	(0.906, 5.407)	0.991	(0.332, 2.957)	0.793	(0.248, 2.538)
Walking speed (m/s)								
≥1.0 m/s	1		1		1		1	
<1.0 m/s	0.262	(0.104, 0.658)	0.214	(0.078, 0.591)	0.089	(0.022, 0.360)	0.104	(0.026, 0.426)
Hemoglobin (g/dL)								
Normal range	1		1		1		1	
Below normal range	1.093	(0.473, 2.528)	0.936	(0.358, 2.447)	0.616	(0.217, 1.744)	0.739	(0.250, 2.185)
ASMI (kg/m^2^)								
Normal	1		1		1		1	
Low ASMI	7.846	(2.853, 21.577)	6.783	(2.416, 19.040)	3.703	(1.067, 12.857)	1.954	(0.445, 8.581)
Grip strength (kg)								
Normal	1		1		1		1	
Low grip strength	1.194	(0.504, 2.831)	0.905	(0.358, 2.287)	0.876	(0.319, 2.406)	0.727	(0.249, 2.117)

Data were analyzed by a logistic regression. Model 1 was adjusted for age and sex, model 2 was adjusted for age, sex, and baseline body-mass index (BMI), and model 3 was adjusted for age, sex, baseline BMI, and baseline appendicular skeletal muscle index (ASMI). SOF, the study of osteoporotic fractures; MNA-SF, Mini-Nutritional Assessment Short Form. Normal range of calf circumference, Males > 34 cm, Females > 33 cm; normal range of hemoglobin, Males 12.3~18.3 g/dL, Females 11.3~15.3 g/dL; normal range of ASMI, Males ≥ 7.0 (kg/m^2^), Females ≥ 5.7 (kg/m^2^) [21]; normal range of grip strength, Males ≥ 28 kg, Females ≥ 18 kg.

## Data Availability

Data available on request due to privacy/ethical restrictions.

## Supplementary Materials

The following supporting information can be downloaded at: https://www.mdpi.com/article/10.3390/nu15194291/s1, Supplementary Table S1. Effects of nutritional supplement drink (NSD) on body composition, physical performance, nutritional status, and nutrition intake in older nursing home residents at risk of malnutrition; Supplementary Table S2. Effects of nutritional supplement drink (NSD) and nutritional education (NE) on blood biochemical parameters in older nursing home residents at risk of malnutrition; Supplementary Table S3. Effects of nutritional supplement drink (NSD) on the MOS 36-Item Short Form Health Survey (SF)-36 questionnaire of older nursing home residents at risk of malnutrition; Supplementary Table S4. Changes in the MOS 36-Item Short Form Health Survey (SF)-36 questionnaire score after 12 weeks of nutritional supplement drink (NSD) in older nursing home residents at risk of malnutrition; Supplementary Table S5. Correlations of components of the MOS 36-Item Short Form Health Survey (SF)-36 questionnaire with the nutritional status, physical performance, vitamin D, and nutritional supplement drink (NSD); Supplementary Table S6. Correlations of albumin levels with muscle mass, physical function, frailty, and nutrition status.
