# Supplementary material for: Beneficial Effects of Oral Nutrition Supplements on the Nutritional Status and Physical Performance of Older Nursing Home Residents at Risk of Malnutrition
Source: Nutrients. 2023 Oct 8;15(19):4291. doi: 10.3390/nu15194291 (PMC10574690; doi:10.3390/nu15194291)
Supplement: Supplementary file 1 [file nutrients-15-04291-s001.zip › nutrients-2639152-supplementary.pdf]

Supplementary Table S1. Effects of nutritional supplement drink (NSD) on body composition, physical performance, nutritional status, and nutrition intake in older nursing home residents at risk of malnutrition

	NSD (*N*=57)						NE (*N*=50)					
				p value						*p* value		
	Baseline	6th week	End	Baseline× 6th week	6th week × End	Baseline × End	Baseline	6th week	End	Baseline × 6th week	6th week × End	Baseline × End
**Body composition**												
Body weight (kg)	53.73 ± 8.31	54.73 ± 8.28	54.94 ± 8.50	0.002	0.513	<0.001	62.91 ± 13.57	63.07 ± 13.47	62.73 ± 12.67	0.304	0.200	0.574
BMI (kg/m^2)	20.47 ± 3.03	20.84 ± 3.20	20.97 ± 3.03	0.013	0.375	<0.001	23.74 ± 4.30	23.81 ± 4.29	23.67 ± 3.98	0.252	0.211	0.603
Body fat (%)	25.97 ± 5.92	26.25 ± 6.54	26.58 ± 6.13	0.413	0.490	0.273	27.03 ± 5.08	27.12 ± 4.42	27.43 ± 4.16	0.786	0.456	0.356
Muscle mass (%)	26.31 ± 3.03	26.19 ± 3.19	26.15 ± 3.13	0.381	0.840	0.499	26.81 ± 2.81	26.62 ± 2.93	26.49 ± 2.92	0.145	0.667	0.313
ASMI (kg/m^2)	5.38 ± 0.95	5.45 ± 0.94	5.46 ± 0.97	0.123	0.799	0.189	6.38 ± 1.38	6.35 ± 1.40	6.30 ± 1.33	0.272	0.494	0.263
Calf circumference (cm)	31.98 ± 3.40	32.18 ± 2.78	32.14 ± 3.01	0.299	0.529	0.842	32.95 ± 4.05	32.76 ± 4.15	32.57 ± 3.91	0.025	0.343	0.065
**Physical performance**												
SOF	0.98 ± 0.64	0.46 ± 0.63	0.23 ± 0.42	<0.001	0.001	<0.001	0.50 ± 0.61	0.44 ± 0.54	0.56 ± 0.58	0.083	0.014	0.180
Grip strength (kg)	21.66 ± 6.14	22.78 ± 6.11	21.97 ± 6.21	0.002	0.109	0.481	21.64 ± 8.09	21.69 ± 7.48	21.88 ± 7.74	0.916	0.51	0.621
6-m walking speed (s)	10.02 ± 5.04	9.20 ± 4.65	8.56 ± 4.79	0.005	0.020	0.001	7.71 ± 2.12	8.28 ± 2.57	8.16 ± 2.89	0.061	0.722	0.111
Walking speed (m/s)	0.73 ± 0.30	0.79 ± 0.31	0.89 ± 0.38	0.004	0.003	<0.001	0.83 ± 0.20	0.78 ± 0.19	0.83 ± 0.32	0.076	0.425	0.363
**Blood pressure**												
SBP (mmHg)	133 ± 17	137 ± 16	133 ± 15	0.121	0.021	0.857	132 ± 17	129 ± 14	131 ± 14	0.102	0.355	0.251
DBP (mmHg)	78 ± 11	79 ± 9	76 ± 8	0.218	0.002	0.079	77 ± 10	76 ± 9	76 ± 9	0.459	0.791	0.574
**Nutritional status**												
MUST												
0, *n* (%)	0 (0.0)	27 (47.4)	34 (59.6)	<0.001	0.4651	<0.001	0 (0.0)	1 (1.9)	2 (3.8)	0.522	0.703	0.355
1, *n* (%)	37 (64.9)	15 (26.3)	12 (21.1)				41 (78.8)	41 (78.8)	40 (76.9)			
2, *n* (%)	10 (17.5)	14 (24.6)	9 (15.8)				9 (17.3)	7 (13.5)	8 (15.4)			
3, *n* (%)	10 (17.5)	1 (1.8)	2 (3.5)				0 (0.0)	1 (1.9)	0 (0.0)			
MNA-SF	9.07 ± 1.83	11.12 ± 1.67	12.04 ± 1.31	<0.001	<0.001	<0.001	10.66 ± 1.92	10.82 ± 1.75	10.58 ± 1.93	0.021	0.247	0.773

Total energy intake (kcal)	1213.9 ± 69.53	1607.4 ± 72.62	1611.8 ± 71.21	<0.001	0.166	<0.001	1219.0 ± 67.68	1221.0 ± 56.32	1223.0 ± 59.08	0.796	0.564	0.417

Data are expressed as the mean ± standard deviation. The Shapiro–Wilk test was used to determine the normality of the population. Data were compared by a paired *t*-test, *t*-test, or Wilcoxon signed-rank test. NE, nutritional education; BW, body weight; BMI, body-mass index; ASMI, appendicular skeletal muscle index; SOF, study of osteoporotic fractures; MUST, malnutrition universal screening tool; MNA-SF, Mini-Nutritional Assessment Short Form.

Supplementary Table S2. Effects of nutritional supplement drink (NSD) and nutritional education (NE) on blood biochemical parameters in older nursing home residents at risk of malnutrition

	NSD						NE					
				p value						*p* value		
	Baseline	6th week	End	Baseline × 6th week	6th week × End	Baseline × End	Baseline	6th week	End	Baseline × 6th week	6th week × End	Baseline × End
Blood sugar (AC) (mg/dL)	107.42 ± 49.78	115.16 ± 40.87	114.79 ± 51.82	0.041	0.791	0.166	109.78 ± 49.35	116.42 ± 45.75	110.21 ± 31.79	0.004	0.151	0.089
**Lipid profile**												
Cholesterol (mg/dL)	163.33 ± 27.05	159.90 ± 23.74	164.39 ± 28.28	0.127	0.082	0.694	170.62 ± 40.86	167.86 ± 38.30	164.56 ± 40.87	0.758	0.359	0.224
Triglyceride (mg/dL)	107.56 ± 77.30	117.60 ± 80.65	116.07 ± 78.39	0.109	0.996	0.269	107.72 ± 52.60	124.46 ± 74.57	139.79 ± 93.51	0.057	0.174	0.001
**Kidney function**												
Uric acid (mg/dL)	5.70 ± 1.52	5.68 ± 1.73	5.63 ± 1.59	0.821	0.696	0.517	6.16 ± 1.92	6.08 ± 1.73	6.03 ± 1.82	0.458	0.512	0.763
Creatinine (mg/dL)	1.09 ± 0.33	1.01 ± 0.32	1.07 ± 0.34	0.002	<0.001	0.31	1.16 ± 0.75	1.18 ± 0.82	1.23 ± 0.96	0.365	0.197	0.28
**Liver function**												
AST (U/L)	27.10 ± 10.21	28.69 ± 9.19	28.89 ± 8.68	0.087	0.494	0.015	28.34 ± 18.15	29.80 ± 16.63	28.11 ± 11.79	0.136	0.414	0.92
ALT (U/L)	18.35 ± 10.13	21.04 ± 10.18	23.30 ± 12.43	0.001	0.014	<0.001	22.86 ± 26.58	21.22 ± 11.18	21.75 ± 11.92	0.113	0.886	0.271
**Nutritional status**												
Albumin (g/dL)	4.28 ± 0.27	4.18 ± 0.32	4.19 ± 0.32	0.002	0.726	0.02	4.17 ± 0.41	4.21 ± 0.42	4.15 ± 0.47	0.286	0.309	0.756
**Vitamin D status**												
Total 25-OH Vit D (ng/mL)	23.93 ± 9.43	23.17 ± 8.64	23.69 ± 9.56	0.067	0.197	0.821	21.83 ± 9.09	21.52 ± 8.71	21.03 ± 8.02	0.38	0.22	0.082
**Zinc status**												
Zinc (μg/L)	750.28 ± 169.61	695.72 ± 97.62	730.09 ± 146.99	0.017	0.042	0.846	829.42 ± 170.44	778.34 ± 143.48	711.10 ± 142.16	0.024	<0.001	<0.001
**Hematology**												
RBCs (10^6/μL)	4.27 ± 0.59	4.27 ± 0.56	4.29 ± 0.60	0.987	0.658	0.748	4.40 ± 0.73	4.34 ± 0.76	4.41 ± 0.73	0.116	0.119	0.804
WBCs (10^3/μL)	6.71 ± 3.77	6.87 ± 5.19	7.48 ± 6.76	0.925	0.004	0.044	6.44 ± 1.74	6.59 ± 1.60	6.52 ± 1.60	0.375	0.66	0.687
Hemoglobin (g/dL)	12.94 ± 1.69	12.88 ± 1.70	12.85 ± 1.84	0.545	0.734	0.484	13.04 ± 2.17	12.93 ± 2.20	12.98 ± 2.16	0.319	0.744	0.681
Hematocrit (%)	38.90 ± 4.84	39.17 ± 4.67	39.42 ± 5.15	0.364	0.378	0.174	40.12 ± 6.27	39.85 ± 6.36	40.06 ± 6.12	0.425	0.67	0.902
Platelets (10^3/μL)	225.70 ± 72.70	226.32 ± 78.39	232.70 ± 77.63	0.760	0.173	0.184	229.36 ± 73.39	227.57 ± 72.76	227.76 ± 70.47	0.653	0.97	0.8

MCH (pg)	30.54 ± 3.27	30.35 ± 3.22	30.13 ± 3.27	0.022	0.021	0.002	29.86 ± 2.81	29.97 ± 2.74	29.60 ± 2.58	0.051	0.001	0.049
MCHC (g/dL)	33.01 ± 1.14	32.87 ± 1.13	32.56 ± 1.10	0.100	0.009	<0.001	32.19 ± 1.22	32.41 ± 1.09	32.34 ± 1.10	0.012	0.768	0.151
MCV (fL)	92.37 ± 8.11	92.22 ± 8.23	92.43 ± 8.33	0.862	0.765	0.562	92.64 ± 6.91	92.33 ± 6.77	91.41 ± 6.79	0.056	<0.001	0.001
Neutrophil Seg. (%)	59.89 ± 10.98	59.60 ± 11.13	61.32 ± 12.69	0.694	0.120	0.047	58.67 ± 9.97	58.85 ± 9.49	59.06 ± 9.33	0.731	0.838	0.717
Lymphocytes (%)	29.74 ± 10.80	29.82 ± 10.83	28.03 ± 11.54	0.745	0.081	0.035	29.96 ± 9.03	29.74 ± 8.43	30.04 ± 8.76	0.682	0.72	0.866
Monocytes (%)	6.77 ± 1.59	6.82 ± 1.61	6.84 ± 1.53	0.687	0.731	0.954	6.65 ± 1.60	6.67 ± 1.48	6.67 ± 1.61	0.981	0.992	0.762
Eosinophils (%)	2.95 ± 2.41	3.12 ± 2.69	2.83 ± 2.43	0.378	0.209	0.559	4.14 ± 5.25	4.14 ± 3.99	3.64 ± 2.55	0.096	0.823	0.261
Basophils (%)	0.66 ± 0.32	0.64 ± 0.30	0.64 ± 0.37	0.434	0.456	0.146	0.58 ± 0.32	0.60 ± 0.30	0.59 ± 0.32	0.269	0.816	0.854
RDW-CV (%)	13.85 ± 1.36	13.79 ± 1.37	13.76 ± 1.29	0.181	0.874	0.254	13.81 ± 1.94	13.68 ± 1.75	13.56 ± 1.65	0.089	0.907	0.25

Data are expressed as the mean ± standard deviation. The Shapiro-Wilk test was used to test determine the normality of the population. Data were compared by a paired *t*-test, *t*-test, or Wilcoxon signed-rank test.

ALT, alanine aminotransferase; AST, aspartate aminotransferase; MCH, mean corpuscular hemoglobin; MCHC, mean corpuscular hemoglobin concentration; MCV, mean corpuscular volume; RBCs, red blood cells; TC, total cholesterol; TIBC, total iron-binding capacity; WBCs, white blood cells; RDW-CV: red blood cell distribution width.

Supplementary Table S3. Effects of nutritional supplement drink (NSD) on the MOS 36-Item Short Form Health Survey (SF)-36 questionnaire of older nursing home residents at risk of malnutrition.

	NSD (*N*=50)							NE (*N*=39)							NSD × NE	
	Baseline			End			*p* value Baseline × End	Baseline			End			*p* value Baseline × End	*p* value Baseline	*p* value End
PF	76.83	±	21.36	84.50	±	18.47	0.001	86.41	±	3.24	85.25	±	3.80	0.020	0.328	0.009
RP	67.22	±	16.21	69.38	±	17.55	0.345	73.08	±	4.57	73.72	±	2.93	0.392	0.061	0.306
BP	87.96	±	14.92	92.96	±	10.96	0.061	96.21	±	7.05	94.62	±	8.35	0.359	0.006	0.614
GH	57.90	±	15.74	61.94	±	13.98	0.059	49.54	±	4.52	49.21	±	4.53	0.800	0.002	<0.001
VT	62.25	±	14.99	69.34	±	18.00	0.007	55.13	±	4.27	54.17	±	5.61	0.392	0.005	<0.001
SF	78.75	±	19.60	83.50	±	17.21	0.057	87.50	±	6.41	85.90	±	7.13	0.236	0.068	0.813
RE	69.79	±	17.67	73.00	±	11.49	0.368	74.36	±	4.00	73.93	±	3.91	0.157	0.145	0.451
MH	65.10	±	13.42	71.60	±	16.64	0.003	59.10	±	3.60	59.49	±	3.40	0.637	0.022	<0.001
PCS	51.23	±	6.09	53.03	±	5.04	0.015	54.04	±	1.33	53.67	±	1.22	0.201	0.011	0.511
MCS	45.77	±	7.52	48.12	±	7.00	0.020	43.69	±	1.27	43.57	±	1.47	0.497	0.026	0.002

Data are expressed as the mean ± standard deviation. The Shapiro-Wilk test was used to determine the normality of the population. Data were compared by a *t*-test, Mann-Whitney U test, paired *t*-test, or Wilcoxon signed-rank test. NE, nutritional education; PF, physical functioning; RP, role limitations of physical problems; BP, bodily pain; GH, general health; VT, vitality; SF, social functioning; RE, role emotional; MH, role emotional; PCS, physical component score; MCS, mental component score.

Supplementary Table S4. Changes in the MOS 36-Item Short Form Health Survey (SF)-36 questionnaire score after 12 weeks of nutritional supplement drink (NSD) in older nursing home residents at risk of malnutrition.

	NSD (*N*=50)			NE (*N*=39)			NSD × NE
	Δ week 12 - baseline			Δ week 12 - baseline			*p* value
PF	7.67	±	14.23	-1.15	±	2.92	<0.001
RP	3.50	±	22.84	0.64	±	4.92	0.170
BP	5.00	±	17.86	-1.59	±	10.20	0.053
GH	4.04	±	14.79	-0.33	±	5.21	0.128
VT	7.09	±	17.53	-0.96	±	6.65	0.011
SF	4.75	±	17.84	-1.60	±	8.20	0.011
RE	6.00	±	23.27	-0.43	±	1.86	0.089
MH	6.50	±	13.41	0.38	±	3.87	0.002
PCS	1.81	±	4.84	-0.37	±	1.77	0.005
MCS	2.35	±	6.90	-0.12	±	1.63	0.018

Data were calculated by the value of week 12 – the baseline and are expressed as the mean ± standard deviation. The Shapiro-Wilk test was used to determine the normality of the population. Data were compared by a *t*-test or Mann-Whitney U test. NE, nutritional education; PF, physical functioning; RP, role limitations of physical problems; BP, bodily pain; GH, general health; VT, vitality; SF, social functioning; RE, role emotional; MH, role emotional; PCS, physical component score; MCS, mental component score.

Supplementary Table S5. Correlations of components of the MOS 36-Item Short Form Health Survey (SF)-36 questionnaire with the nutritional status, physical performance, vitamin D, and nutritional supplement drink (NSD).

	MNA-SF		BMI		Grip strength		Walking speed		Albumin		Vitamin D		NSD	
	r	*p* value	r	*p* value	r	*p* value	r	*p* value	r	*p* value	r	*p* value	r	*p*- value
PF	0.371	<0.001	0.111	0.302	0.145	0.175	0.493	<0.001	0.114	0.286	-0.029	0.787	0.278	0.008
RP	-0.089	0.408	-0.090	0.403	0.151	0.158	0.079	0.462	0.013	0.907	-0.099	0.354	-0.109	0.309
BP	0.029	0.787	0.036	0.739	-0.132	0.217	0.055	0.609	0.105	0.326	-0.169	0.113	-0.054	0.617
GH	0.334	0.001	-0.076	0.477	0.256	0.015	0.300	0.004	0.235	0.026	0.046	0.668	0.528	<0.001
VT	0.299	0.004	-0.052	0.627	0.071	0.507	0.113	0.292	0.027	0.799	0.046	0.670	0.457	<0.001
SF	-0.084	0.436	-0.090	0.401	0.014	0.894	0.138	0.198	-0.016	0.884	0.047	0.662	0.025	0.815
RE	-0.123	0.251	-0.186	0.081	0.048	0.658	0.093	0.389	0.127	0.235	0.008	0.942	0.080	0.454
MH	0.271	0.010	-0.095	0.377	0.002	0.988	0.065	0.547	0.051	0.632	0.096	0.372	0.374	<0.001
PCS	0.141	0.187	0.098	0.363	0.132	0.218	0.479	<0.001	0.106	0.322	-0.156	0.144	0.070	0.514
MCS	0.065	0.546	-0.162	0.129	-0.040	0.710	-0.049	0.646	-0.005	0.964	0.127	0.236	0.332	0.001

Data were analyzed by Spearman's rank correlation and were compared to each item in the SF-36 questionnaire (*N*=89). BMI, body-mass index; MNA-SF, Mini-Nutritional Assessment Short Form; PF, physical functioning; RP, role limitations of physical problems; BP, bodily pain; GH, general health; VT, vitality; SF, social functioning; RE, role emotional; MH, role emotional; PCS, physical component score; MCS, mental component score.

Supplementary Table S6. Correlations of albumin levels with muscle mass, physical function, frailty, and the nutrition status.

	Baseline		6th week		End	
	r	*p* value	*r*	*p* value	*r*	*p* value
Calf circumference	0.345	<0.001	0.260	0.007	0.289	0.003
ASMI	0.289	0.002	0.185	0.056	0.190	0.050
Grip strength	0.304	0.001	0.023	0.815	0.242	0.012
Walking speed (m/s)	0.361	<0.001	0.192	0.047	0.210	0.030
SOF	-0.019	0.848	0.077	0.429	0.157	0.107
MNA-SF	0.221	0.022	0.160	0.101	0.233	0.016

Data were analyzed by Spearman's rank correlation and were compared to the albumin level (*N*=107). ASMI, appendicular skeletal muscle index; SOF, study of osteoporotic fractures; MNA-SF, Mini-Nutritional Assessment Short Form.
